# Achieving Health Equity for Older Adults Through State-of-the-Art Innovations

**DOI:** 10.1093/geroni/igaa057.2287

**Published:** 2020-12-16

**Authors:** Karen Fortuna, John Batsis, Daniel Jimenez

## Abstract

As health indicators such as life expectancy have improved for many older adults, some older adults experience a disproportionate amount of preventable disease, death, and disability. The causes of health disparities among older adults are multidimensional in that disparities are due to multiple, interacting factors such as socioeconomic status, disability status, geographic location, and race/ethnicity. Achieving health equity in late-life requires innovative strategies to address interconnected environmental, sociocultural, behavioral and biological factors that impede opportunities to achieve optimal health and quality of life. This symposium will present state-of-the-art innovations and strategies employed among socially disadvantaged racial, ethnic, and other population groups, and communities. We will discuss innovations in the workforce enhancements with older adult peer support specialists and community health workers, community engagement techniques in program design, and digital solutions aimed at addressing multiple dimensions of health in older adults.

